# Studies on the Two-Step Aging Process of Fe-Based Shape Memory Single Crystals

**DOI:** 10.3390/ma13071724

**Published:** 2020-04-07

**Authors:** Monika Czerny, Grzegorz Cios, Wojciech Maziarz, Yuri Chumlyakov, Robert Chulist

**Affiliations:** 1 Institute of Metallurgy and Materials Science, Polish Academy of Sciences, 30059 Krakow, Poland; w.maziarz@imim.pl (W.M.); r.chulist@imim.pl (R.C.); 2 Academic Centre for Materials and Nanotechnology, AGH University of Science and Technology, Al. Mickiewicza 30, 30059 Krakow, Poland; ciosu@agh.edu.pl; 3 Siberian Physical Technical Institute, Tomsk State University, 634050 Tomsk, Russia; chum@phys.tsu.ru

**Keywords:** B2 precipitates, single crystal, Fe-based shape memory alloys, coherency, martensitic transformation

## Abstract

Fe_50_Ni_28_Co_17_Al_11.5_Ta_2.5_ single crystals oriented along the [001] direction were investigated in order to establish the influence of two-step aging conditions on superelastic properties. The homogenized and quenched single crystalline material was subjected to a combination of high-temperature and low-temperature heat treatment at 973 K for 0.5 h and at 723 K for various aging times, respectively. As a result, fine and coherent γ’ precipitates were formed. Using diffraction of high energy synchrotron radiation, the volume fraction of γ’ precipitates was computed while their size was determined by high resolution TEM analysis. Compared with one-step heat treatment, the two-step aging process enables control of the precipitate size in a more accurate way. Moreover, it allows one to obtain a higher volume fraction of precipitates without increasing their size significantly. The obtained coherent γ’ precipitates ranged in size from 5 to 8 nm; that considerably improved mechanical properties. The highest superelastic response was obtained for single crystals aged at 973 K for 0.5 h followed by aging at 723 K for 3 h. The single crystals treated with such conditions exhibited a superelastic strain of 15% in which the mechanical martensite stabilization was substantially suppressed.

## 1. Introduction

Over the past twenty years there has been a growing interest in new intelligent materials; among them, shape memory alloys (SMA), in which shape change occurs by applying an external thermal, magnetic or mechanical field, have received a great deal of attention. These include Fe-based SMAs that are characterized by low material cost and good formability which may result in broad application [1,2,3,4,5]. However, the main drawback of Fe-based alloys is poor shape recovery and limited superelastic strain due to non-thermoelastic martensitic transformation [6,7,8,9,10,11,12,13,14,15]. This area of research was substantially revived after the publication of Tanaka et al., wherein a huge superelastic strain of over 13.5% in polycrystalline Fe28Ni17Co11.5Al2.5Ta (NCAT) (at.%) samples was reported [16]. The reported superelastic effect, as well as the shape recovery, were linked with a crystallographically reversible thermoelastic martensitic transformation. It is worth noting that this effect only appears in Fe-based SMAs if the coherent precipitates of γ’ phase with the L1_2_-type structure are introduced to the system. Other requirements deal with proper texture and grain size of the material [4,5,6,7,8,9,10,11,12,13,14,15,16,17,18]. On one hand the precipitates strengthen the matrix, but on the other, they act as stress concentrators which stimulate martensitic transformation. However, the size of the precipitates has to be tuned in order to distort the precipitates only elastically. Precipitates being too small or too large can result in dislocation cutting (Friedl mechanism) or in the formation of Orowan loops around the precipitates. Both mechanisms are associated with plastic deformation which lowers the resistance to cyclic degradation [18,19,20,21,22,23]. Therefore, it is of great interest to find an optimal heat treatment which would allow one to control the precipitate size, their distribution and the volume fraction [24,25,26,27,28,29,30,31,32,33,34,35,36,37,38,39,40,41,42,43,44,45].

In our recent study we reported the effect of a one-step aging process on the precipitation behavior, including phase morphology, chemical composition and degree of coherence in Fe-28Ni-17Co-11.5Al-2.5Ta single crystals [46]. The particles were investigated with transmission and scanning electron microscopies along with high energy X-ray diffraction. The single crystalline material of [100] (001) orientation was subjected to three different heat treatments: slowly cooled, quenched and then annealed with various aging conditions. These measurements were followed by mechanical testing. The best mechanical properties and the highest completely reversible superelastic strain were obtained for the samples treated at 973 K for times ranging between 0.5 and 1 h. Therefore, to further explore the most interesting aging time, two-step aging was performed. This process, as a combination of two heating operations, involves the use of heat treatment at 973 K for only 0.5 h and low-temperature aging at 723 K for longer times. Such a strategy is typically used to strengthen the Al 2xxx series alloys, wherein dislocations interact with the so-called GP zones in the form of nanoscale inclusions [47,48,49,50,51]. 

In this paper, we study the effects of two-step aging on the microstructure and mechanical properties of Fe-28Ni-17Co-11.5Al-2.5Ta single crystals with [100] (001) crystal orientation. The main goal is to control the effective size and volume fraction of γ’ in order to obtain fully reversible superelastic response and to reduce the mechanical stability [46,52,53,54]. The abovementioned stability is relatively large for this group of alloys and it is typically linked to the shift of austenite start (As) temperature towards a higher temperature.

## 2. Materials and Methods 

The single crystals with the nominal composition Fe_41_Ni_28_Co_17_Al_11.5_Ta_2.5_ were grown by the Bridgman method [46]. The material was subsequently homogenized at 1573 K for 48 h in a vacuum furnace. The single crystalline samples were sealed in quartz ampules and heated at 1573 K for 1 h followed by water quenching. To determine the crystallographic orientation and chemical homogeneity a SEM FEI Quanta 3D (FEI, Eindhoven, Netherlands) well-fitted with energy dispersive X- ray spectrometer (EDS) produced by EDAX and TSL EBSD system (EDAX, Mahwah, NJ, USA) were applied. The prepared single crystals were cut along [100] (001) orientation into cuboid samples with dimensions of 3 × 3 × 10 mm^3^. The re-measured EBSD orientation shows accuracy better than 1°. Subsequently, the homogenized samples were aged at 973 K for 0.5 h and then the aging treatment was conducted at 723 K for different times, 1, 2 and 3 h. The phase analysis was performed using high energy synchrotron X- ray radiation at DESY Hamburg Germany using beam line P07B (87.1 keV, λ = 0.0142342 nm). The beam size was 0.8 × 0.8 mm^2^. The resulting X-ray transmission mode allowed obtaining diffraction information from large sample volumes. In addition to providing a good statistic and to eliminate the effect of texture, the samples were continuously rotated about the ω-axis from −90° to +90° during the measurements [55,56]. In such a way, all diffraction spots were integrated into one 2theta/intensity plot yielding a “powder-type diffraction.” The chemical composition and microstructure were investigated using a Tecnai G2 TEM (FEI, Eindhoven, Netherlands) operating at 200kV refit with an energy dispersive X- ray (EDX) microanalyser (EDAX, Mahwah, NJ, USA) and a high angle annular dark field detector (HAADF). The thin foils were prepared with electrolyte consisting of 20% perchloric acid and 80% ethanol at a temperature of about 250 K. Before observation, the thin samples were ion-milled using a Leica EM RES 101 (Fremont, USA). The sizes of the precipitates were measured using the HAADF-STEM (Fischione, Pennsylvania, Pittsburgh, PA, USA) images. Mechanical testing of the single crystals along the [100] direction was performed using a Instron 5966 machine in liquid nitrogen (77 K) and at a strain rate of 2 × 10^−4^.

## 3. Results and Discussion

**Figure 1:**
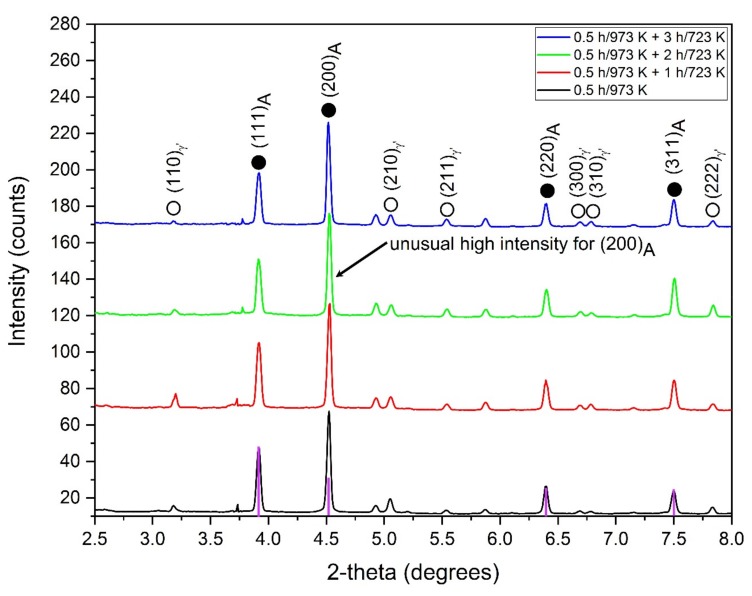
Synchrotron X-ray diffraction patterns of the annealed NCAT samples of <001> orientation aged at 973 K for 0.5 h and then aged at 723 K for variable times. The purple bars indicate the intensities generated by a powder diffraction of the matrix (theoretical diffraction).

Large, brittle and non-coherent β precipitates have no relation with the crystal structure of the surrounding matrix. Consequently, they are practically undeformable and the main strengthening mechanism is related to their size and distribution due to dislocation bypass [46]. This in turn, affects only the dislocation destiny and results in high work hardening but does not promote the superelastic effect. For this reason the β (NiAl-type) precipitates are undesired in NCAT alloys. As shown in Figure 1, an efficient quenching leads to complete suppression of the β phase [46]. Only the austenitic matrix and coherent γ’ precipitates (Ni_3_Al-type) can be distinguished in the diffraction pattern. Similarly to the previous study, a very high intensity of the (200) reflection compared to other ones is detected. 

**Figure 2:**
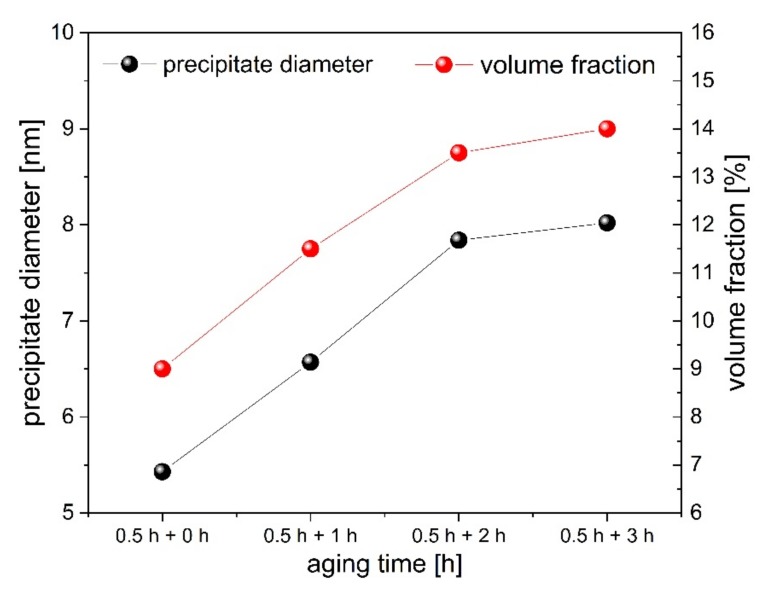
Precipitate diameter and volume fraction of γ’ as a function of secondary aging (low temperature). The first time regards aging at 973 K, whereas the second regards aging at 723 K.

Generally, this could be attributed to a texture effect since we are dealing with single crystalline material; however, the applied methodology provides a powder-type diffraction excluding the effect of texture. For this reason the enormous high intensity of the (200)_A_ peak is observed as a real effect and should be included in the discussion. This is additionally supported by the fact that other reflections, such as (111)_A_, (220)_A_ and (311)_A_ correspond very well with theoretical intensities. For better clarity the theoretical intensities of all reflections were computed and presented with respect to (111) planes for the single crystal aged at 973 K and for 0.5 h in Figure 1 (purple bars). Interestingly, the (200)/(111) peak intensity ratio continues to grow with increasing time of low-temperature aging. For the single crystal aged at 973 K for 0.5 h it is about 1.48, while for the single crystal aged at 973 K for 0.5 h and 3 h at 723 K it reaches 1.98. Such an observation may indicate a more rectangular/disk shape of the particles and/or very high ordering, coherence or degree of periodicity for the {200} planes. This can be also used to tune the mechanical properties, since the higher the ratio, the better the superelastic response that can be observed for the given single crystal. The quantity of the γ’ phase computed based on the Rietveld refinement is approximately equal to 9% for the single crystal aged at 973 K for 0.5 h [46] and it increases constantly to about 14% upon further aging for 3 h at 723 K. However, both the precipitate size and their volume fraction appear to reach a saturation value since the curves become straight lines that decrease in slope, Figure 2. This situation is very different compared to that observed in a one-step aging process, wherein the amount of γ’ phase and the average size steadily increase, even up to 29% and 15.5 nm for 10 h of aging at 973 K [46].

**Figure 3:**
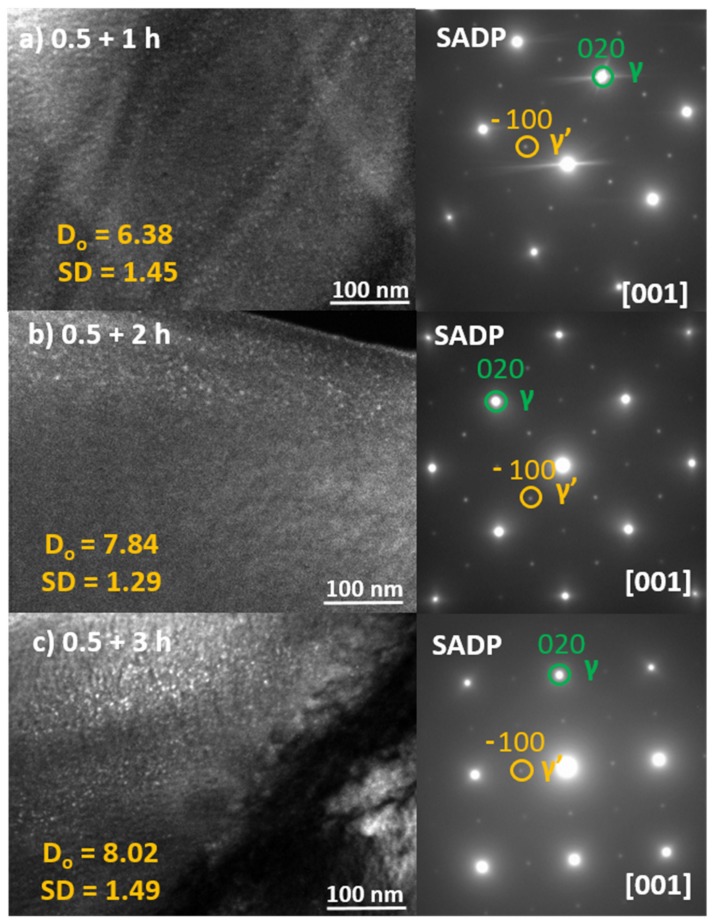
DF TEM microstructures and the corresponding selected area electron diffraction patterns showing the γ’ precipitates in the FeNiCoAlTa single crystal aged at 973 K and for 1, 2 and 3 h at 723 K. D_o_—average precipitate diameter (nm), SD—standard deviation of precipitate diameter (nm).

TEM investigations shown in Figure 3 demonstrate dark field (DF) images and the associated selected area diffraction patterns (SADP) taken from the samples annealed at 973 K for 0.5 h and 1, 2 and 3 h at 723 K. As can be seen, the average γ’ size increases as the time of low temperature aging increases. It starts with approximately 5 nm for the one-step aged single crystal [46] and grows up to about 8 nm for 3 h of aging at 723 K. The diameter was calculated using image analyses performed on DF (dark field) images obtained from (100) reflections of γ’ phase presented in Figure 3. All DF images presented here represent the thin areas neighboring the hole of the thin foil in order to ensure the same conditions of acquisition. The image analysis was conducted using the Gatan DigitalMicrograph (Pleasanton, CA, USA) collecting at least 25 particles.

**Figure 4:**
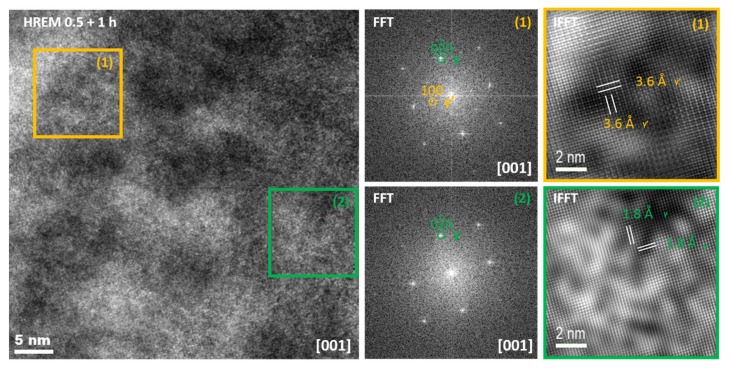
HRTEM image and corresponding FFT and IFFT images of the material aged at 973 K for 0.5 h and 1 h at 723 K.

All samples measured show a very good coherency between the austenitic matrix and the γ’ precipitates. This can be clearly seen in Figure 1 using the diffraction of high synchrotron radiation, since no splitting of the main reflections can be detected in X-ray diffraction pattern. The same holds for high resolution (HRTEM) images illustrated for instance in Figure 4. This HRTEM image and the corresponding numerical diffraction pattern using fast Fourier transform (FFT) along with inverse FFT demonstrate two different areas. In the first region a precipitate indicated by extra (100) reflection can be seen, while in the second, only precipitate-free matrix can be detected. The same applies to the corresponding IFFT image which clearly demonstrates an additional spacing with 3.6 Å for (100) plane. 

In the next step the single crystals were subjected to compression tests. The aged single crystals deformed at room temperature showed only plastic deformation; therefore, the compression tests were performed at 77 K. It should be mentioned that at 77 K the single crystals still exhibit an austenitic structure. The maximum theoretical strain calculated for the given crystallography (using lattice parameters of austenite and martensite) amounts to 8.7% and 14.1% for tension and compression, respectively [57,58]. To study the effect of aging condition on the superelastic strain, only untrained single crystals with the same dimensions and crystallographic orientation were tested, unless stated otherwise. This was intended to avoid the training effect, which is rather complex for this group of alloys, especially if we consider stabilization that demands heating and cooling cycles.

**Figure 5:**
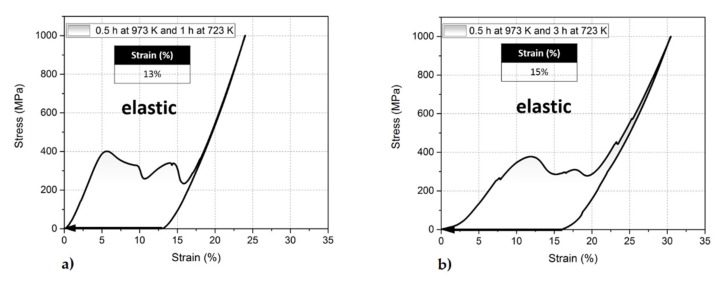
Superelastic response of the [001] oriented FeNiCoAlTa single crystal aged at 973 K and 723 K for (**a**) 0.5 + 1 h (**b**) 0.5 + 3 h under compression at 77 K (first cycle).

The stress–strain curves, presented in Figure 5, measured for samples aged with a two-step process (second aging at 723 K for 1 h and 3 h) show a superelastic strain of about 15%. This amount of strain is in good agreement with the theoretical value and indicates that upon compression all precipitates transform together with the matrix. Generally, similar character of stress–strain curves can be obtained for one-step and two-step aging. Both display characteristic two humps with the first one related to the onset of martensitic transformation and the second one to reorientation of martensitic variants. 

However, comparing these two approaches, the two-step aging process significantly decreases the stress for the onset of martensitic transformation from about 500 MPa in the one-step aging (0.5 h at 973 K) to about 400 MPa for two–step aging (0.5 and 1 h at 723 K). This value goes further down to about 380 MPa for single crystals aged for 3 h at 723 K. Thus, the combination of low and high-temperature aging reduces the stress for the onset of martensitic transformation by about 120 MPa. Nevertheless, samples subjected to about 1 GPa show the so-called martensite stabilization effect. It means that the superelastic strain which at 77 K seems to be permanent, goes first back if the samples are heated up to a higher temperature. As already described in [46] this effect is strongly related to the single variant state and the deficiency of austenite/twinned martensite interfaces. 

**Figure 6:**
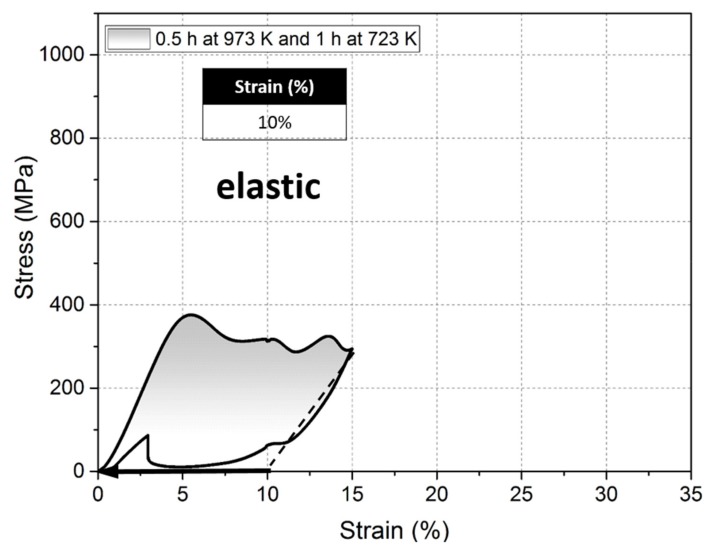
Superelastic response of the [001] oriented FeNiCoAlTa single crystal aged at 973 K for 0.5 h and 723 K for 1 h under compression at 77 K (second cycle).

To transform back to austenite it requires an addition energy demand to form twin variants and austenite/twinned martensite interface. This magnitude of overheating seems to be correlated with twining stress, as the higher the twinning stress, the higher the degree of stabilization that can be monitored [52,53,54]. However, this study indicates that this effect can be considerably reduced if a proper size and distribution of particles are introduced to the alloy. For example Figure 6 shows a fully reversible strain of about 10% obtained at 77 K which is twice as large as that obtained with one-step aging [46]. This stress–strain curve also shows a training effect, since the stress for the onset of martensitic transformation decreases by about 20 MPa when applying a second cycle.

Thus, based on the previous experiments, we propose two-step aging process which allows one to control precipitation hardening in a more accurate way. The aging process is divided into two steps; i.e., higher temperature for short time and lower temperature for longer time. Such a procedure allows one to obtain a higher volume fraction of precipitates without increasing their size significantly. This in turn, lowers the stress for the onset of martensitic transformation. The best mechanical properties are strictly related with an unusual high intensity of (200) reflections, which cannot be observed if the aging at 973 K is longer than 0.5 h [46]. Despite the fact that this effect is not completely understood, it can be used to find the optimum between annealing conditions and mechanical properties in order to achieve the best superelastic response in NCAT alloys. It was also observed that two-step aging decreases the effect of martensite stabilization. This study reveals one more important issue. Unlike one-step (high temperature) aging, the two-step aging process allows one to reach the saturation values for precipitate size and their volume fraction at a lower level and in a more controlled way. These values seem to be strongly correlated with primary aging (temperature and time) which sets the kinetic conditions for low temperature aging. However, to comprehend the effect of primary aging (high temperature) on the kinetics of secondary aging (low temperature) in Fe-based single crystals, further study is needed. 

## 4. Conclusions

In summary, it can be concluded that two-step aging, which combines short-time high-temperature and longer time low-temperature aging, effectively decreases the stress for the onset of martensitic transformation. This process facilitates controlling the size and distribution of γ’ in the nanoscale range in a very accurate way. The obtained coherent γ’ precipitates range in size from 5 to 8 nm. Single crystals treated under such conditions exhibit a fully reversible superelastic strain of about 15% in which the mechanical martensite stabilization is considerably suppressed. Additionally, an extremely high intensity of (200) reflection was detected for all annealed samples. This intensity increases with increasing time of low-temperature aging, which is correlated with better superelastic properties.
